# Contemporary advances in polymer applications for sporting goods: fundamentals, properties, and applications

**DOI:** 10.1039/d4ra06544a

**Published:** 2024-11-26

**Authors:** Qingyao Li, Iftikhar Ahmed, Phan Minh Ngoc, Ta Phuong Hoa, Tran Vinh Dieu, Muhammad Sultan Irshad, Ho Xuan Nang, Van-Duong Dao

**Affiliations:** a Xi'an Aeronautical Institute Sports Department China 201602001@xaau.edu.cn; b College of Health Sciences, Abu Dhabi University P.O. Box 59911 Abu Dhabi United Arab Emirates Dr.Iftikhar.Ahmed@live.fr; c Faculty of Biotechnology, Chemistry and Environmental Engineering, Phenikaa University Hanoi 100000 Vietnam dieu.tranvinh@phenikaa-uni.edu.vn duong.daovan@phenikaa-uni.edu.vn; d Ministry of Education Key Laboratory for the Green Preparation and Application of Functional Materials, Collaborative Innovation Center for Advanced Organic Chemical Materials Co-constructed by the Province and Ministry, School of New Energy and Electrical Engineering, Hubei University Wuhan 430062 P. R. China muhammadsultanirshad@hubu.edu.cn; e Faculty of Vehicle and Energy Engineering, PHENIKAA University Hanoi Vietnam nang.hoxuan@phenikaa-uni.edu.vn

## Abstract

Polymers have transformed sportswear, bringing forth a new age of innovation. Because of these flexible materials, athletes in many disciplines benefit from lighter, more durable gear. Polymers significantly improve sports performance, ranging from carbon-fiber tennis rackets that increase power to cushioning polymers that reduce joint impact in running shoes. This review provides a basic understanding of polymers, classifications, and their potential role in sporting goods. It also explores lightweight design, impact resistance, and even smart sports gear. Further, the fabrication procedures of various polymer matrix composites have been explored for sporting goods applications. However, this work briefly discussed the challenges and limitations associated with polymers in sports goods, including cost considerations, durability, longevity, and regulatory compliance. It provides insight into future developments in this field as well as the multifaceted role of polymers in sports goods.

## Abstract 2

Polymers enhance sports goods, offering lightweight, durable, and eco-friendly designs. This review explores the types, applications, and future trends of polymers while addressing challenges like cost and durability.

## Graphical abstract

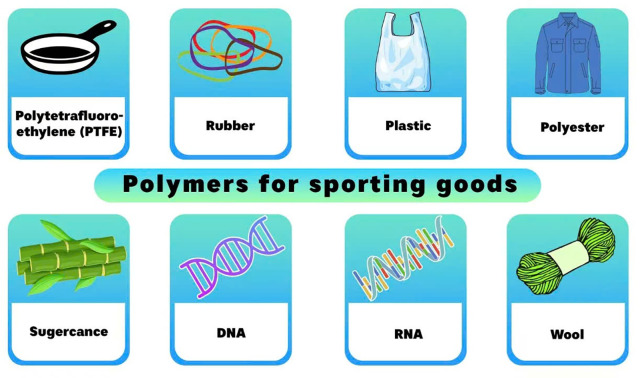

## 1. Introduction

Sports serve as a window into scientific and technological progress, reflecting the accomplishments of a country in these fields. The improvement of athletes' performance is significantly influenced by developments in material technology.^1–5^ Modern sports evolved from natural, straightforward tools and activities to high-tech, cutting-edge tools, facilities, and activities.^6^ The majority of sporting goods in the past were constructed of wood and metal, which were cumbersome, heavy, and poorly designed. There is now sporting equipment that is sturdy, lightweight, and long-lasting thanks to new polymer materials.^5,6^ The performance of athletes has been aided by the use of sports apparel composed of breathable material for simple respiration and temperature regulation.^7,8^ Sports clothing uses an amino acid polymer coated with nylon taffeta that is also waterproof.^8^ Every sport demands equipment that has been specifically made for it. The choice of materials is crucial to produce sporting goods that meet the required criteria.^9^ Depending on the sport in question, materials that have all the desired qualities are frequently made from a mix of several materials, incorporating composites, polymers, metals, and ceramics.^10–12^ Materials for sporting goods are evaluated according to their usability, strength, weight, water resistance, and other qualities. It is critical in sports to have supportive, portable, durable, and long-lasting gear that enhances athletes' performance.^13,14^ Modern polymers improve comfort, convenience, homogeneity, utility, and consistency in sporting equipment when compared to conventional materials.^1,2,13,14^ A polymer matrix surrounds statistically dispersed gas bubbles in a two-phase system known as polymer foam.^15^ Products made of foamed polymers have several desirable qualities, such as low density, excellent energy absorption (impact resistance), and good heat and sound insulation.^16^ These advantages help to explain why they are used in a variety of applications. The consumption of polymer foams, which totals more than 26 million tons annually,^17–19^ is a clear sign of the market's continued growth. These foams are widely used in a variety of industries, including those that make vehicles, buildings, packaging, and sports equipment.^14,16,17^

In the field of sports goods applications, polymers play a significant and multifaceted role, helping to improve the performance, safety, and design of various sports equipment and gear.^20,21^ Due to their distinctive combination of qualities, including their lightweight nature, durability, flexibility, and resistance to impacts and weather conditions, these high-performance materials are frequently used.^16,20,22,23^ As a result, polymers have completely changed the sports business, enabling the creation of innovative goods that satisfy the various demands of sportsmen and sports aficionados. A healthy lifestyle and participation in sports have always been important to people.^4,5,7^ Education has increased people's understanding of the importance of good cleanliness and physical fitness. Numerous lifestyle-related disorders, including diabetes, hypertension, obesity, high/low blood pressure, and others, are prevented by physical activity.^24,25^ Additionally, it promotes children's physical fitness and wellness. Their cardiovascular systems and lungs get stronger, and their bones become healthier and stronger.^24,25^

Sports serve as a window into scientific and technological progress, reflecting the accomplishments of a country in these fields. The improvement of athletes' performance is significantly influenced by developments in material technology.^1,2^ Modern sports evolved from natural, straightforward equipment and activities to high-tech, advanced materials, equipment, and locations. Equipment design is one of the most important fields in material science and engineering.^26,27^ The continual discovery of innovative materials has had a significant impact on the production of highly effective sporting goods. Modern sports equipment has emerged as a result of advancements in material technology.^27^ Tennis, where graphite fiber-reinforced polymers are used, golf, where tungsten-weighted golf clubs are used, and vaulting poles, where glassy metal inlays are used, all exhibit notable advantages.^28^ These examples show the advantages of using cutting-edge materials. While improving the efficiency, comfort, and appearance of sporting products. The advantages of novel materials over traditional materials include their light weight, weight-to-strength ratio, corrosion resistance, and other characteristics.^7,10^ The rigidity and strength of these new materials are superior to those of previous ones. The innovative materials are also found to have enhanced hardness and torsion strain resistance.^10,29,30^ These features are used in many pieces of athletic equipment. There is a lot of research being done to develop new materials for athletic products on a global scale.^5^ A variety of materials, including wood, twine, gut, rubber, sophisticated metal-matrix composites, ceramics, polymers, and synthetic hybrid materials are used to make sports equipment.^7^

Materials may be found everywhere in sports. For different sports, a variety of materials are required, some of which are organically occurring and others of which are artificial or synthetic.^31^ The sources of natural resources are plants and animals. Two forms of textiles made from plants and trees are silk and cotton.^8^ Humans have developed many polymers, glass, plastics, and other synthetic materials.^16^ Plastic, a byproduct of the distillation of crude oil, is the most common synthetic material.^12^ The preferred polymeric material for molded sports products is polypropylene (PP) which combined with fiber glass is often used in sports equipment such as skis, snowboards, and tennis rackets. This equipment is lightweight, strong, and durable and enables athletes to improve their performance.^32^ A typical polymer used to create sporting items is acrylonitrile–butadiene–styrene^33^ such as helmets, protective gear, and even some parts of sports equipment.

Herein, this review delves into the transformative impact of polymers on the world of sports equipment. Polymers have spearheaded a paradigm shift in this industry, ushering in an era marked by unparalleled innovation and enhancement.^5^ A multitude of athletes, spanning a wide spectrum of disciplines, have reaped the benefits of these versatile materials, owing their efficacy to the intricate chains of repetitive molecular units.^4,34,35^ This molecular architecture has paved the way for the creation of sports gear that is not only lighter but also more enduring, ultimately culminating in higher performance levels.^1^ Take, for example, the evolution of tennis rackets crafted from advanced carbon-fiber composite polymers, which have significantly amplified power and control.^36–38^ Likewise, running shoes now feature cushioning polymers that mitigate the impact on joints, thereby reducing the risk of injury.^4^ Polymers have indeed become instrumental in elevating athletic prowess. Beyond performance, they have also played a pivotal role in fostering environmental sustainability within the sports equipment domain. Their inherent recyclability and repurposing capabilities have significantly curtailed the ecological footprint associated with sports equipment production.

The relentless advancement of polymer science inspires optimism for the future of sports technology. It promises further enhancements in athletic performance, ensuring a healthier and more sustainable future for sports enthusiasts across the globe. This comprehensive review embarks on an exploration of the indispensable role that polymers play in the design, manufacturing, and performance augmentation of sports goods. The journey commences with a foundational understanding of polymers and their pivotal significance in sports equipment. Subsequently, the review traverses through the various categories of polymers commonly deployed in sports goods, encompassing thermoplastics, thermosetting materials, elastomers, and composite structures.^39,40^ Each section meticulously examines the applications of these polymers in the context of specific sports equipment, such as tennis and badminton rackets, golf clubs, running shoes, soccer balls, and cycling helmets. Intricate aspects of polymer technology, such as lightweight design, impact resistance, energy absorption, flexibility, and elasticity, are scrutinized. The discourse extends to futuristic prospects and innovations within the sports equipment industry, including the integration of smart technology, customization options, bio-inspired materials, and wearable tech. Amidst these remarkable advancements, the review candidly addresses the challenges and limitations associated with polymers in the realm of sports goods. These encompass considerations of cost, durability, longevity, and compliance with regulatory standards. In offering this multifaceted perspective, the review not only sheds light on the imminent developments in this field but also underscores the intricate and multifarious role that polymers continue to play in the realm of sports goods.

### 1.1 Polymers

Any category of compounds, whether organic or synthetic, is composed of macromolecules, which are oversized versions of monomers, the more fundamental chemical building blocks.^41^ Proteins, cellulose, and nucleic acids are only a few examples of polymers that are found in living organisms. Additionally, they act as the building blocks for human-made materials including concrete, glass, paper, plastic, and rubber as well as minerals like feldspar, quartz, and diamond.^41,42^

**Fig. 1:**
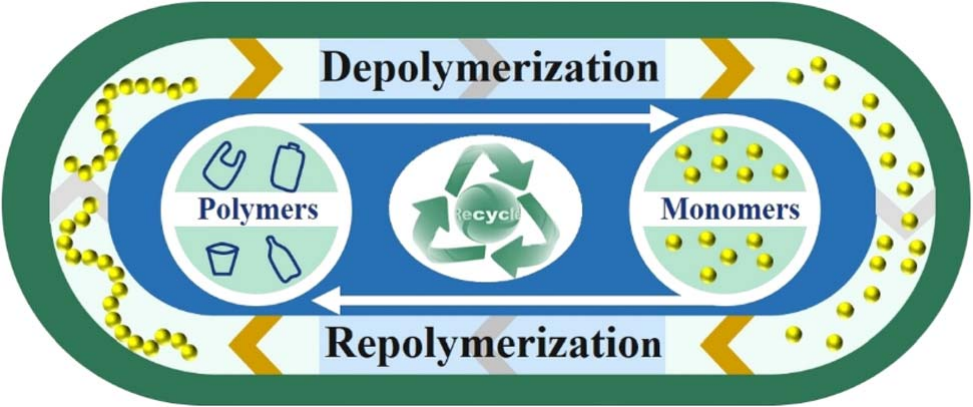
Cyclic conversion of monomer to polymer and *vice versa*.^44^ Copyright 2023, Wiley.

Polymer is a vital component of numerous important industrial goods. In addition to social factors, the necessity to replace conventional materials is what is driving their rapid development in production. The word “polymer” was first used by Swedish chemist J. J. Berzelius. For instance, he thought that benzene (C_6_H_6_) is a polymer of ethane (C_2_H_2_).^43^ Later, this definition underwent a small adjustment. Plastics, natural and synthetic fibers, rubber, coatings, adhesives, sealants, and other materials that are all now very common are all the subject of the relatively new field of polymer science.^44^ Cyclic conversion of monomers and polymers allows materials to change structural states, increasing flexibility, recyclability, and sustainability. This reversible transformation is critical in the development of self-healing materials, adaptive polymers, and eco-friendly plastics, as well as stimulating material science innovation for applications that need resilience and environmental awareness as demonstrated by the cyclic conversion in Fig. 1.^44^ The mixture is occasionally referred to as a high polymer when there are numerous monomers present. Polymers don't need to develop from monomers with the same molecular weight, shape, or chemical composition. Some natural polymers only contain one kind of monomer.^43,44^ However, the bulk of polymers, both natural and artificial, are copolymers, made up of two or more different types of monomers. Sports equipment has evolved as a result of the development of new and cutting-edge materials.^16^ The performance of sportsmen is significantly improved by sports equipment with smart designs.

It is possible to separate polymers into two groups. Examples of natural polymers, commonly referred to as biopolymers, include silk, rubber, cellulose, wool, amber, keratin, collagen, starch, DNA, and shellac.^45^ Biopolymers have crucial functions in organisms as functional proteins, structural polysaccharides, structural proteins, nucleic acids, and molecules that store energy.^46^ Synthetic polymers are produced routinely in laboratories by chemical processes. Several examples of synthetic polymers are PVC (polyvinyl chloride), nylon, silicone, polyethylene, and polystyrene.^47^ Synthetic polymers are used to create a variety of daily items, including plastics, adhesives, paints, and mechanical parts. Synthetic polymers can be divided into two groups.^48^ A liquid or soft solid can be permanently changed into an insoluble polymer by heating or irradiating the raw material for thermoset plastics. Thermoset polymers are frequently rigid and have high molecular weights.^49^ When plastic is twisted, it maintains its warped shape and frequently disintegrates before melting. Thermoset plastics include substances like polyester, epoxy, acrylic resins, vinyl esters, and vinyl.^48,49^ Thermoset plastics also come in Bakelite, Kevlar, and vulcanized rubber varieties.^50^

**Fig. 2:**
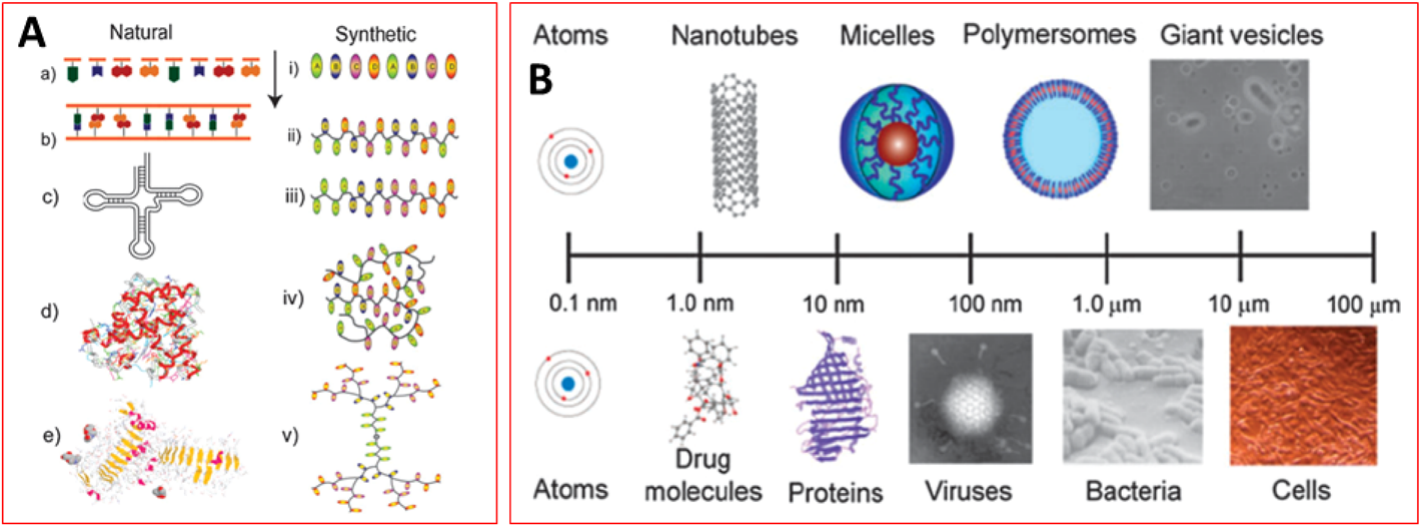
(A) Structures and length scale of natural and synthetic polymers.^54^ (B) Systems with increasing complexity in terms of length scales and structures/architectures.^54^ Copyright 2010, Royal Society of Chemistry.

**Fig. 3:**
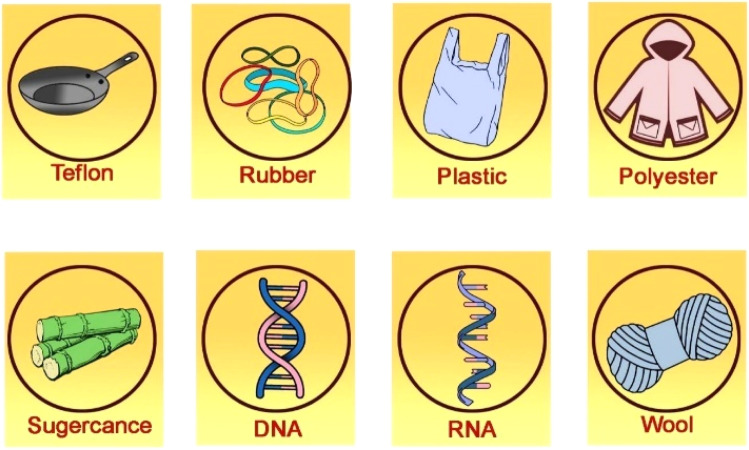
Examples of different polymers.

Thermoplastic polymers, also referred to as thermo-softening plastics, are the other class of synthetic polymers.^51,52^ In contrast to Thermoset plastics' stiffness, thermoplastic polymers can be molded above a certain temperature and become solid when they cool.^51^ While the chemical linkages in thermoset plastics are irreversibly formed when they are cured, they decrease with temperature in thermoplastics.^53^ George, *et al.*^54^ demonstrate the comparison of natural and synthetic approaches toward polymer synthesis and their controlled morphologies for diverse applications. The controlled assembly of synthetic polymer structures is now possible with an unprecedented range of functional groups and molecular architectures as illustrated in Fig. 2. Thermoplastics melt into a liquid upon heating as opposed to thermoset, which disintegrates instead of melting. Nylon (PTFE), Teflon (PA), polyethylene, polypropylene, polycarbonate, acrylic, and polycarbonate are a few examples of thermoplastics.^53^ There are different polymer examples such as demonstrated in Fig. 3.

### 1.2 Importance of polymers in sports goods

Today, a wide range of polymer materials are used in a variety of sports. The desire for healthy competition and enhanced performance among athletes grows as more people take up sports, and they continue to look for high-quality, durable, practical, yet affordable sporting goods.^31,45,48^ This chapter discusses the various ways that polymers can be used in sports, particularly in the design of sporting goods like garments and footwear.^1,6–8^ The most popular polymers for a range of sporting applications are covered in this chapter. Sports use polymers for a variety of purposes.^54^

**Fig. 4:**
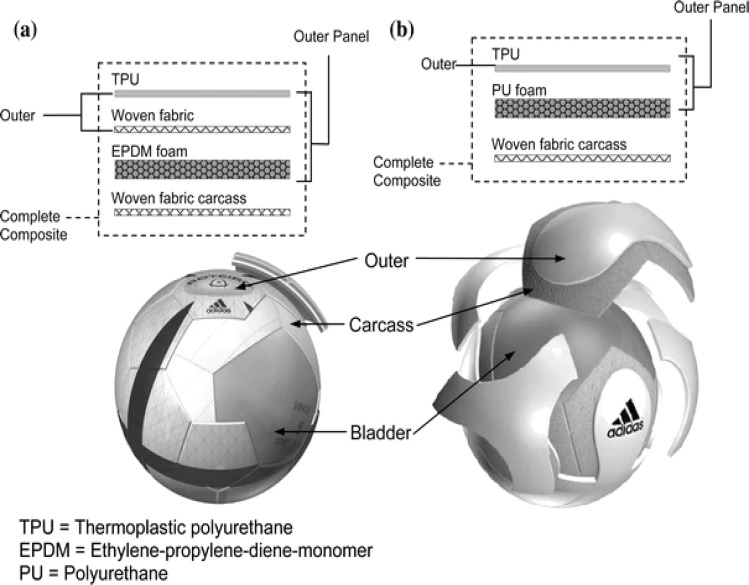
(a and b) Soccer balls are based on multi-layer textile-reinforced polymer composites.^55^ Copyright 2008, Springer.

Price *et al.*^55^ reported the viscoelasticity of multi-layer textile-reinforced polymer composites used in soccer balls. Polymers have a large and diverse role in the realm of sports goods applications, helping to enhance the functionality, security, and aesthetics of numerous sporting goods, as illustrated in Fig. 4.^55^ Due to their distinctive combination of qualities, including their lightweight nature, durability, flexibility, and resistance to impacts and weather conditions, these high-performance materials are frequently used.^55,56^ As a result, polymers have completely changed the sports business, enabling the creation of innovative goods that satisfy the various demands of sportsmen and sports aficionados.^57^ Polymer composite ball utilized is more prevalent than the standard ball in the table tennis starting, receiving, and holding phases, which greatly raises athletes' scores.^58^ Thus, it can be concluded that the predictions of the scoring rate for players of table tennis in the starting, receiving, and holding stages of the field, based on the polymer composite ball, are satisfactory. Polymers have revolutionized the sports sector by supplying materials that improve functionality, security, and aesthetics in a variety of applications for sporting goods.^57,58^ Continuous advancements in polymer science research and development help to provide cutting-edge gear that improves athletes' performance while also improving the general enjoyment and safety of sports aficionados throughout the world.

## 2. Various polymer types used in sporting goods

**Fig. 5:**
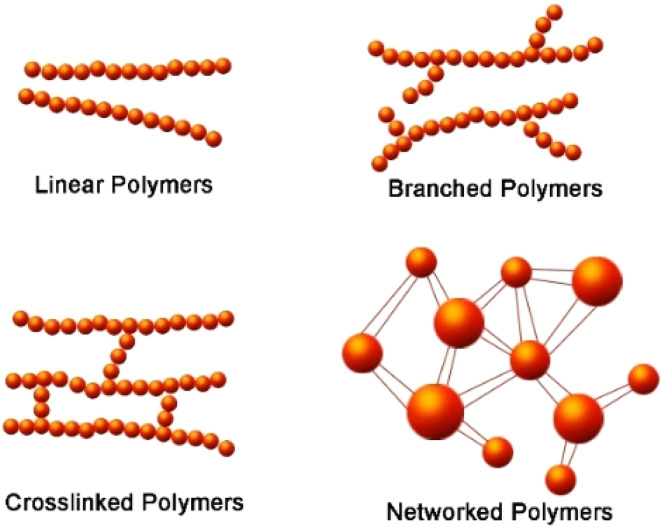
Types of polymer structures are dependent on the topological structure differences.

A wide range of polymers are used in the manufacture of sporting goods to suit the requirements of various sports and equipment.^32,43,59–63^ Polymers can be divided into three categories: linear polymers, branch-chain polymers, and cross-link polymers, dependent on the topological structure differences as illustrated in Fig. 5.

**Fig. 6:**
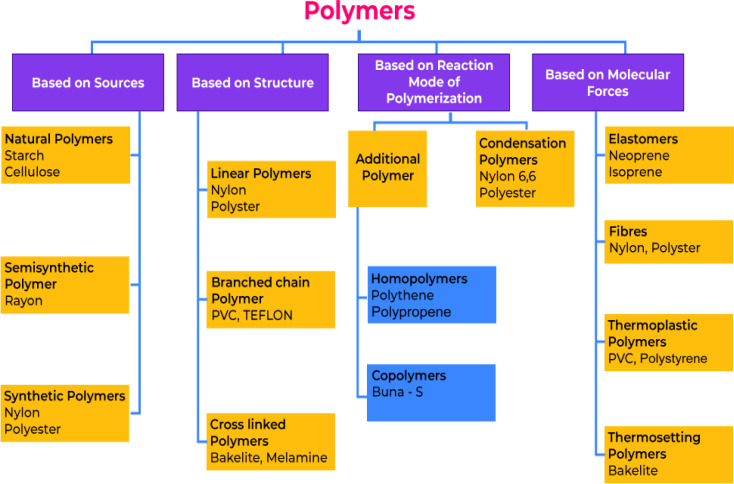
The classification of polymers is illustrated in the chart below.^64–68^

The other two categories of polymers can be distinguished based on the polymerization reaction used to create them *e.g.*, addition polymerization, and condensation polymerization.^60–62^ It is also possible to categorize polymers in two ways based on the monomers used to create them *e.g.* homomer, and heteromer or co-polymer. A polymer may be classified into either elastomers, fibers, thermoplastics, or thermosetting polymers depending upon the sources, structures, reaction ode of polymerization, and molecular forces between their constituent atoms.^64–68^Fig. 6 demonstrates the detailed classification of polymers based on different conditions.

The importance of thermoplastics lies in their versatility, recyclability, and cost-effectiveness, while thermosets are valued for their high-temperature resistance, durability, and chemical resistance.^68^ The choice between these two types of polymers depends on the specific requirements of the application, and often, a combination of both may be used to achieve the desired properties. This review overviews the role of thermoplastics, thermosets, elastomers, and polymer composites in sports applications.

### 2.1 Thermoplastics polymers

**Fig. 7:**
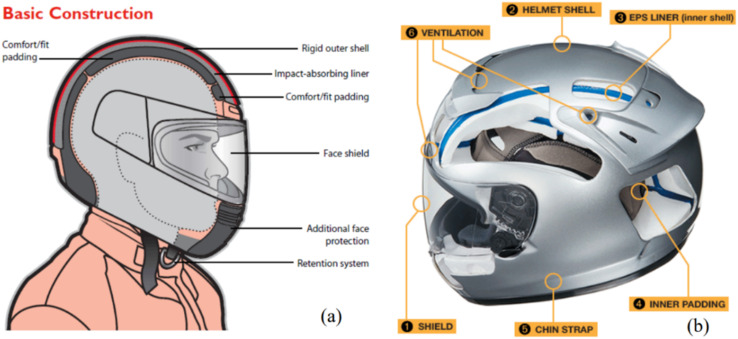
Schematic diagrams showing the various components in a (a) typical motorcycle helmet and (b) a Shoei motorcycle helmet.^71^

A class of polymers known as thermoplastics is distinguished by their propensity to repeatedly melt and reconfigure themselves without significantly altering their chemical composition.^69^ Due to this quality, they are incredibly adaptable and appropriate for a range of commercial and consumer uses, including sporting products. Thermoplastics may be repeatedly molded, cooled, and remolded, unlike thermosetting polymers which suffer permanent chemical changes upon curing, enabling effective manufacturing processes and personalization.^70^ Thermoplastics, such as polyethylene (PE), polypropylene (PP), acrylonitrile–butadiene–styrene (ABS), polycarbonate (PC), polyurethane (PU), polyvinyl chloride (PVC), nylon, polyethylene terephthalate (PET), and thermoplastic elastomers (TPE), offer a myriad of applications within the realm of sporting goods, owing to their advantageous properties.^71–73^ Overall, thermoplastics are indispensable materials in the manufacturing of a wide range of sports products, from footwear and protective gear to sports equipment and accessories. This is due to their versatility, recyclability, and various features, as explained in Table 1. Lai *et al.*^71^ demonstrate the thermoplastic-based motorcycle helmet, as illustrated in Fig. 7.

**Table 1:** Different types of thermoplastic polymers and their role in the sports industry

Thermoset polymer	Formula	Sports goods
Polypropylene (PP)	(C_3_H_6_)_*n*_	Water bottles, sports equipment like helmets, and protective gear due to their lightweight and impact-resistant properties^63,71,74–81^
Polyethylene (PE)	(C_2_H_4_)_*n*_	Plastic bags are used to carry sports gear, as well as in the construction of kayaks and paddleboard hulls^59,82–84^
Polyurethane (PU)	C_27_H_36_N_2_O_10_	Outer shells of rollerblades or inline skates' wheels, as well as cushioning insoles and padding for sports equipment^60,82,85–88^
Polyvinyl chloride (PVC)	(H_2_C–CHCl)_*n*_	Inflatable sports equipment like balls, air mattresses, and pool toys. It's appreciated for its flexibility and resistance to water^62,89–92^
Acrylonitrile–butadiene–styrene (ABS)	(C_8_H_8_·C_4_H_6_·C_3_H_3_N)_*n*_	Construction of helmets and protective gear due to their strength and impact resistance. Components of skateboards and longboards^69,93–96^
Nylon (polyamide)	(C_12_H_22_N_2_O_2_)_*n*_	Racket strings in sports like tennis and badminton, as well as in the construction of sports apparel, including jerseys, shorts, and socks^97–101^
Polycarbonate (PC)	C_15_H_16_O_2_	Protective eyewear for sports like skiing, snowboarding, and cycling^102–106^
Ethylene vinyl acetate (EVA)	(C_2_H_4_)_*x*_·(C_4_H_6_O_2_)_*y*_	Cushioning insoles, midsoles in athletic shoes, and padding in sports equipment like helmets and protective gear^107–111^
Polyethylene terephthalate (PET)	(C_10_H_8_O_4_)_*n*_	Water bottles and other beverage containers used by athletes and sports enthusiasts^112–117^

### 2.2 Thermosetting polymers

The class of polymers known as thermosetting polymers, or thermosets, responds differently to heating. Thermosetting polymers go through a chemical reaction during their initial curing phase that irreversibly turns them into a rigid and infusible state, in contrast to thermoplastic polymers that can be repeatedly melted and reformed.^49,51–53,105^ They cannot be melted or altered once they have been cured without suffering severe deterioration. A type of plastic known as thermosetting polymers, or simply “Thermoset,” is defined by the fact that they are made from a liquid solution that, when heated, transforms irrevocably into a solid.^55,69,70,118^ This chapter focuses on some aspects of the chemistry of epoxy polymers and provides examples of both step-growth and chain-growth polymerizations utilized in the creation of polymer networks. This review explains the structural alterations that occur during the formation of networks, such as gelation and vitrification. After that, processing guidelines for thermosetting polymers are discussed. Regarding the widest range of applications, the next section describes thermosetting polymer processing techniques.^56,119,120^

Predictive simulations of their behavior are becoming more important in understanding the molecular basis of this class of polymers' properties and completing experiments in the pursuit of custom materials for particular applications.^56,119,121,122^ These applications range from structural components for aerospace to electronics packaging. By using all-atom molecular dynamics simulations, it focuses on modeling and simulating the curing process to forecast these polymers' molecular structure and thermo-mechanical response. Vegetable oils make excellent environmentally friendly raw materials for thermosetting polymers. By directly polymerizing triglyceride C=C, and hydrosilylating alkenyl-terminated fatty acid derivatives, organic–inorganic hybrid materials with intriguing properties for optical applications. Vegetable oils are hailed as excellent environmentally friendly raw materials for thermosetting polymers due to their renewable and sustainable nature.^70^ One of the more intriguing chemical processes that produce epoxidized vegetable oils is epoxidation. It describes how epoxidized linseed oil was used to create biobased polyhedral oligomeric silsesquioxanes-nanocomposites.^61,100,120^ In conclusion, because of their distinctive mix of qualities, such as heat resistance, mechanical strength, and chemical resistance, thermosetting polymers are essential in many industries.^100,120^ They are crucial components in situations where durability and high-temperature stability are needed. Thermosetting polymers have some key benefits, including increased resistance to high temperatures, high degrees of dimensional stability, cost-effectiveness, and the capacity for extremely flexible design.^100^ On the other side, some drawbacks of thermosetting polymers include the inability to recycle or reuse the items and the inability to remold or reshape the products, as briefly explained in Table 2.

**Table 2:** Different types of thermoset polymers and their role in the sports industry

Thermoset polymer	Formula	Sports goods
Epoxy resin	1,3-Bis(2,3-epoxypropoxy)-benzene	Surfboards, snowboards, and skis^119,121,123–125^
Fibre-reinforced plastic	Polymer matrix reinforced with fibres	Fishing rods, tennis rackets, and hockey sticks^91,97,126–128^
Phenolic resin	C_8_H_6_O_2_	Fishing reel handles or the grips on golf clubs^122,129–134^
Melamine resin	C_3_H_6_N_6_	It is used in tabletops and surfaces for indoor sports like table tennis or as a protective coating on the top layer of laminate-based sporting equipment^30,129,135–138^
Polyester resin	Unsaturated and saturated dicarboxylic acids	Watercraft, including canoes and kayaks^120,139–142^
Carbon fiber reinforced composites	CFRP	Bicycles, tennis rackets, and golf club shafts^128,139,143–145^
Bakelite	(C_6_H_6_O–CH_2_OH)_*n*_	Tennis racket handles and pistol grips due to their heat resistance and electrical insulating properties^128,146–149^

### 2.3 Elastomers polymers

Elastomers are a group of polymers that are as elastic as rubber and can restore their original shape after being stretched or distorted. They are unique from both thermoplastics and thermosetting polymers and are frequently referred to as rubber materials.^118,130,150^ Elastomers can deform significantly when under stress and then return to their former shape after the load has been removed thanks to their distinctive molecular structure.^130^

Elastomeric biomaterials, such as silicones, thermoplastic elastomers, polyolefin and polydiene elastomers, poly(vinyl chloride), natural rubber, heparinized polymers, hydrogels, and polypeptide elastomers are described in depth.^150,151^ Additionally, reviews are given of transdermal treatment systems, orthotics, prosthetic devices, general medical care products, and ophthalmology-related biomedical applications. Elastomers will be utilized in medical devices more and more due to their biocompatibility, endurance, design flexibility, and favorable performance/cost ratios.^92,118,152–154^ Elastomers will be used extensively in medical technology in the future. Because they may offer elasticity and resistance in a variety of applications, elastomers are essential to numerous industries. They are crucial parts of products that need flexibility, vibration dampening, and shock absorption.^92,152,153^ Rubbers and elastomers are polymers that can change in size in response to stress. When the stress is released, the polymer contracts back to its original size.^92^Table 3 lists the characteristics and uses of a few popular elastomers.

**Table 3:** Different types of elastomer polymers and their role in the sports industry

Elastomer polymer	Formula	Sports goods
Rubber	(C_5_H_8_)_*n*_	Athletic shoes, grips on tennis rackets, basketball, tennis, and golf^155–158^
EVA (ethylene vinyl acetate)	(C_2_H_4_)_*n*_(C_4_H_6_O_2_)_*m*_	Cushioning and shock absorption, running shoes, and other sports footwear^74,159–163^
Silicone rubber	[R_2_SiO]_*n*_	Swimming caps, goggles, and snorkeling masks^151,164,165^
Polybutadiene rubber	(CH_2_=CH–CH=CH_2_)	Golf ball cores^151,166–170^
Nitrile rubber (NBR)	Polymerization of acrylonitrile (CH_2_=CHCN) and butadiene (CH_2_CH–CH=CH_2_)	Scuba diving equipment and inflatable boats^150,171,172^
SBR (styrene–butadiene rubber)	75 percent butadiene (CH_2_=CH–CH=CH_2_) and 25 percent styrene (CH_2_=CHC_6_H_5_)	Treadmill belts and the outer layers of basketballs, soccer balls, and volleyballs^173^
Neoprene	*n*CH_2_=C(Cl)–CH=CH_2_oxygen/benzoylperoxide[–CH_2_–C(Cl)=CH–CH_2_–]_*n*_	Wetsuits for water sports like surfing and diving^72,118,152–154,173–175^

### 2.4 Composite materials polymers

Composite materials are created by combining two or more separate components, each with a unique set of qualities, to produce a material with improved performance characteristics.^61,123^ A polymer matrix and reinforcement materials are frequently combined in the context of polymers to create composites that have particular mechanical, thermal, or electrical properties.^123^ The addition of reinforcing components, which are typically stronger or stiffer than the polymer matrix, helps the composite perform better overall.^123,125^

**Fig. 8:**
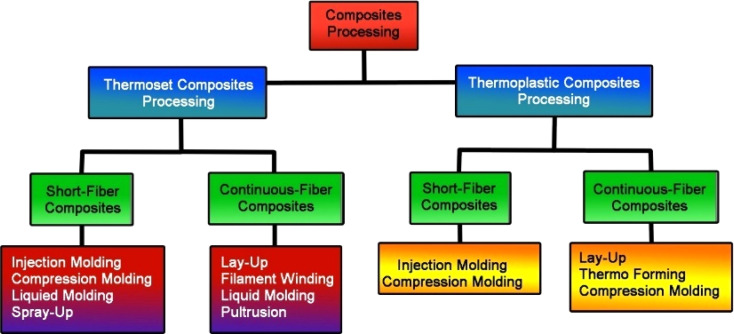
Fabrication procedures of various polymer matrix composites.

What does “composite material” actually mean? An easy way to define a composite material is as follows: When two (or more) materials are fused to create a third substance, such material is referred to as a composite material.^125,143^ Even if this definition is accurate, further consideration shows that it is still far too broad because it implies that almost any material can be classified as a composite.^143^ For instance, the nominal composition of the 2024 aluminum alloy is 93.5% Al, 4.4% Cu, 0.6% Mn, and 1.5% Mg. The 2024 alloy might be considered a “composite” following the broad definition provided above because it is composed of four separate elements (aluminum, copper, manganese, and magnesium) that are atomically bound to one another.^30,125,143^ In a similar vein, almost all metal alloys, polymers, and ceramics satisfy this broad definition of a composite because they all contain more than one sort of constituent atom.^122^ A composite material is a system of materials comprised of two (or more) distinct materials that are linked at the atomic and/or molecular levels but separate at length scales greater than around 1 × 10 m (1 m).^73^ As a result, when composite materials are examined under an optical microscope, it is straightforward to distinguish between the many constituent pieces (or different material phases) that make up composite materials.^51^ In structural composites, a relatively low-strength, low-stiffness matrix material is often enclosed by a high-strength, high-stiffness reinforcing material. In a perfect world, the reinforcing and matrix materials combine to produce a composite that has advantages over either of the two individual constituent materials. Many naturally occurring materials can be thought of as composites.^55^ An excellent example is anything made of wood or laminated wood (Fig. 8).^10^

Composites are increasingly the material of choice wherever expanding technology has produced a need for combinations of attributes that no single material can fulfill.^10^ The creator of a composite can achieve qualities that neither material exhibits on its own by spreading fibers or particles of one component in a matrix, or binder, of another.^10,129^ Already, designers of military and commercial aircraft, sports equipment, and automobiles have turned to composites for some components due to the necessity for rigidity, strength, and low density. Composites are increasingly the material of choice wherever expanding technology has produced a need for combinations of attributes that no single material can fulfill. The creator of a composite can achieve qualities that neither material exhibits on its own by spreading fibers or particles of one component in a matrix, or binder, of another.^10,122,129^ Already, designers of military and commercial aircraft, sports equipment, and automobiles have turned to composites for some components due to the necessity for rigidity, strength, and low density. By carefully balancing the proportions of polymers and reinforcements, composite materials can offer a broad range of qualities. According to the particular needs of the application, such as strength, weight, thermal resistance, and cost, the choice of polymer matrix and reinforcement material is made.^145^ By offering robust yet lightweight substitutes for conventional materials like metals and ceramics, these materials have revolutionized some sectors.^10^ The three basic components used to create composite materials are the matrix, fibers, and additives or fillers.^145^

Polymer composite materials are commonly used in sports equipment. Polymer engineering materials are used in the design and production of many sports facilities and sports equipment to meet various sporting needs, such as an equipment compact structure, heat resistance, corrosion resistance, and longer service life.^128^ Rubber comes in two varieties: natural rubber and synthetic rubber.^135^ In various sports, athletes will experience a significant amount of impact force, which the rubber material may effectively lessen to safeguard the athletes' safety as well as effectively absorb shock and slide.^145^ As a result, various rubber composite materials are frequently employed in the development and manufacture of sporting goods, such as impact-absorbing protection gear and high-elastic sports shoes for landings. When an elastic material is put under external pressure, it deforms reversibly but does not break; once the pressure is released, the rubber returns to its previous shape.^10,55^

## 3. Role of polymers in specific sports goods

The performance, durability, and safety of many sporting goods are enhanced by the use of polymers in their production. Based on their unique qualities and how well they meet the specifications of each piece of sporting equipment, various types of polymers are selected.^43,53,59^ Here are some instances of how polymers are used in particular sports equipment.

### 3.1 Tennis rackets and badminton rackets

Racket frames are created using carbon fiber-reinforced polymers, which are frequently epoxy resin.^58,158,176^ Due to the remarkable strength-to-weight ratios that these polymers offer, lightweight yet robust rackets with enhanced power and control are possible. In tennis, several polymers are employed.^158^ Up until 1965, professional tennis racquets were all constructed of wood. A tennis racket made of steel was invented by a French player named Rene Lacoste in 1965, while the first racket made of aluminum hit the market in 1968.^58^ Metal tennis rackets began to gain popularity over time. The use of metals enabled the creation of new racket designs, such as those with a larger head.^58^

**Fig. 9:**
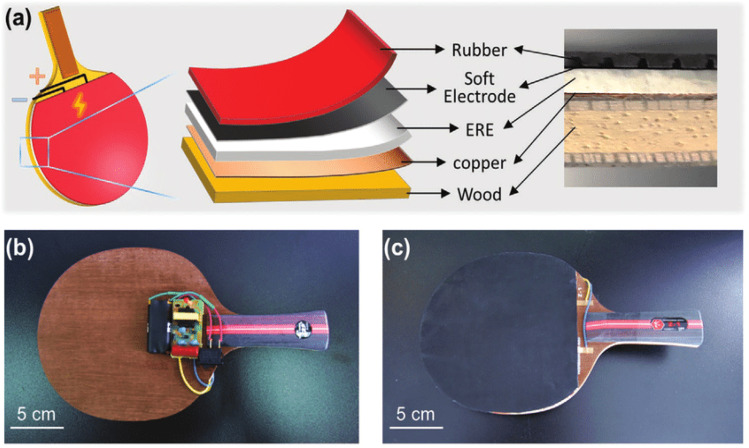
(a–c) Schematic illustration of smart table tennis racket based on anisotropic electrorheological elastomers (EREs).^176^ Copyright 2021, Wiley.

A wooden racket would not play well if the head was built too broadly because this would increase strain. However, because the metal frames were stronger than the wooden ones, they could support more string tension.^158^ The smart table tennis racket demonstrated by Zhou *et al.*^176^ has a customizable stiffness that can be adjusted according to the play style and training method being employed, as demonstrated in Fig. 9. Tennis strings are typically made of polymers; however, some varieties of strings may contain titanium.^58^ The four primary polymeric materials used to make tennis racket strings are nylon, polyester, Kevlar, and natural gut.^158^

### 3.2 Golf clubs

**Fig. 10:**
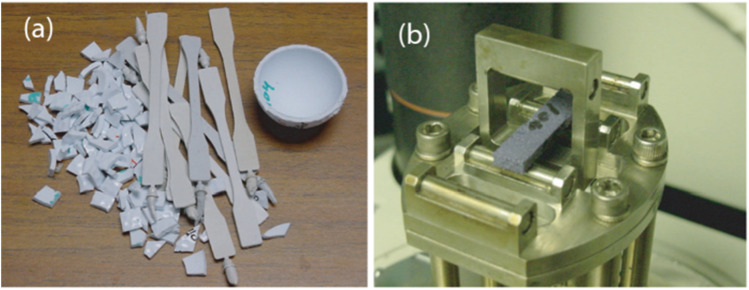
(a) Calibration ball cover, cover material, and tensile test specimens, (b) core flexure specimen in a 3-point bend DMA fixture.^170^

Golf club shafts are made of graphite or carbon fiber composites for flexibility, dampening, and better swing control. Fibers and polymers work together to improve energy transfer during swinging.^169,170^ The majority of golf balls on the market now fall into one of two broad categories: solid or wound. Golf balls with multiple layers, two pieces, and one piece make up solid balls.^168^ Golf balls can be made from a single component with ease. They are also reasonably priced. However, they only provide a few playing qualities. A cover and a solid polybutadiene core make up two-piece golf balls.^166–168^ These have a good range and endure for a long time. A solid core and a cover, either of which may have one or more layers, are often found in golf balls with several layers.^166–168^ Multilayered balls offer a wide range of playing properties despite being more expensive. Wound golf balls contain a fluid-filled center that is encased in an elastomeric material and a cover.^168^ These balls are preferred by golfers over other balls because of their good spin and playing characteristics. Making wrapped core golf balls is expensive and complicated. Mase *et al.*^170^ reported the mechanical properties of golf balls *via* experimental evidence (Fig. 10).

We can adjust the properties of these balls by changing their polymeric compositions or the physical layout of their numerous parts, such as centers and cores.^169^ Golf balls are made from at least one polymeric material plus a compound that might improve impact durability. By combining several monomers, macromolecules known as polymers are produced.^167^ After frequent use and impact, golf balls made of various polymers start to develop microscopic fissures.^168^ Golf balls composed of polymeric material can have their polymer backbone repaired by heating and cooling. The strength of the repaired plastic is equivalent to the strength of the polymeric substance.^66^ Numerous processes can be used to make golf balls.^31^ A golf ball's core, for instance, might be a solid core encased in a cover layer. The core may consist of a single layer or several layers. A sphere filled with fluids or solids and surrounded by an outer core layer could make up the core's center.^166,168,170^

### 3.3 Running shoes

**Fig. 11:**
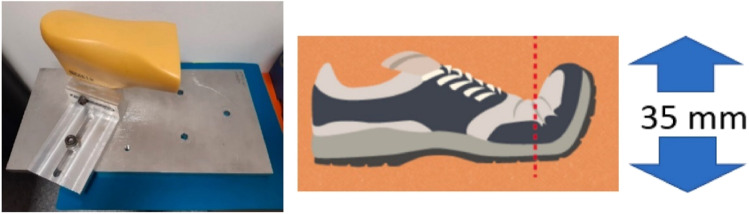
The setup used for the stiffness test. A stiffness test was performed using the Instron 3360 universal testing machine. Footwear was tested with no insole. Distance of extension – 35 mm.^161^ Copyright 2022, Elsevier.

Running shoes frequently use midsoles constructed of polyurethane foam or ethylene-vinyl acetate (EVA).^106,117,162^ As a result of the cushioning and stress absorption these polymers offer, jogging has less of an impact on the feet and joints.^162^ Lunchev *et al.*^161^ reported a universal testing machine from Instron 3360 was used for the stiffness tests of running shoes derived from poly(ethylene vinyl acetate) foams, as illustrated in Fig. 11.^163^ Insoles were not included in the testing of the footwear, and 35 mm is the distance of the extension.^159^

#### 3.3.1 Outsole traction and durability

Running shoes' outsoles are frequently constructed of rubber or synthetic polymers, which offer high traction on a variety of surfaces and are resistant to wear.^98,159^ Modern running shoes frequently have breathable mesh uppers, which are typically polymer-based and designed to enhance comfort and allow air circulation to keep the feet cool.

#### 3.3.2 Support and flexibility

The fabrication of heel counters and overlays uses thermoplastic polyurethane (TPU) or nylon polymers to offer support and stability while preserving flexibility.^85,163^

### 3.4 Soccer balls

**Fig. 12:**
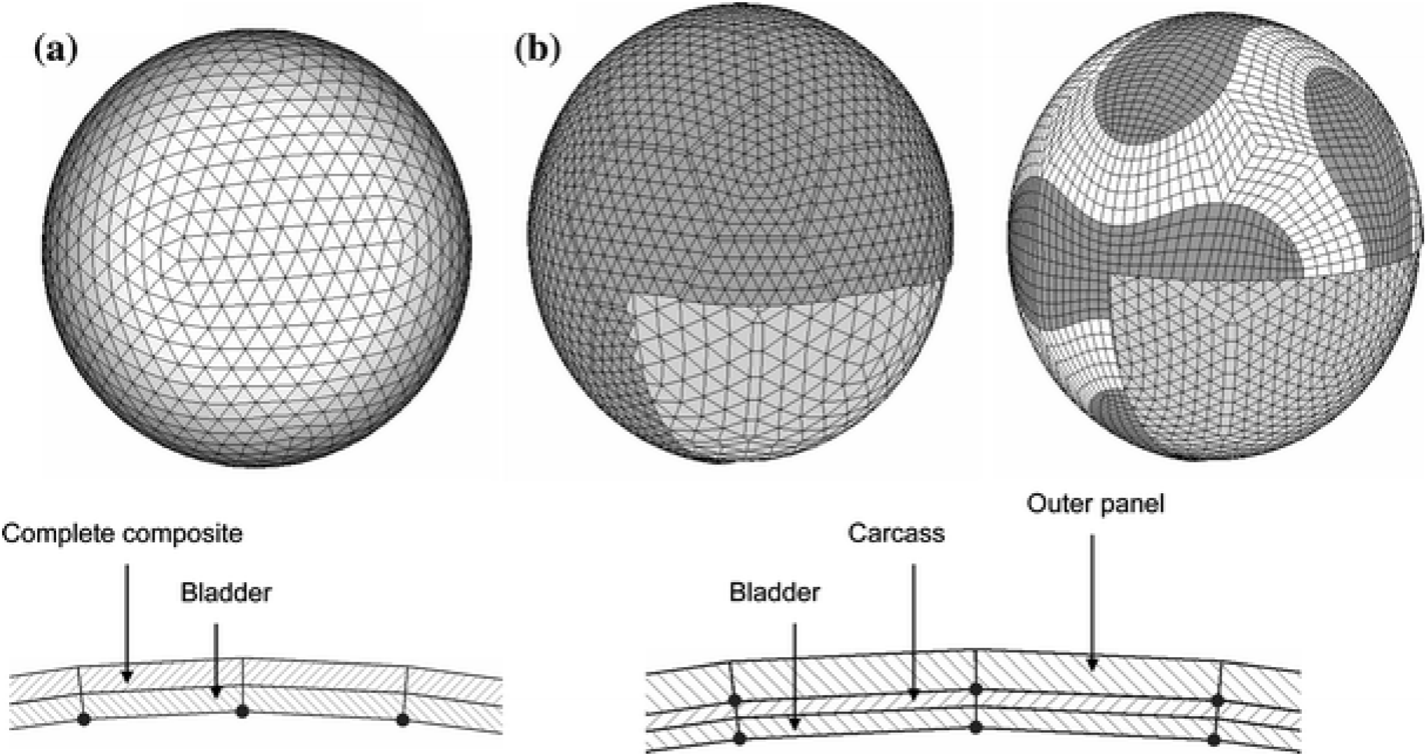
(a and b) Composite soccer balls with multi-layer textile-reinforced polymer composites with high viscoelasticity.^55^ Copyright 2008, Springer.

Soccer balls' outer covers are often constructed of synthetic polymers like thermoplastic polyurethane (TPU) or polyurethane (PU).^54,55^ These substances offer sturdiness, water resistance, and a level playing surface. Soccer balls' inner bladders are frequently made of butyl or latex rubber.^151,154,177^ These components aid in maintaining the ball's form, inflation, and ability to hold air. Soccer balls' panels and shapes are frequently sewn together, and the polymers used for the cover have an impact on the ball's aerodynamics, shape, and overall performance.^25,54,151,154,173,177^ Price *et al.*^55^ reported multi-layer textile-reinforced polymer composites with high viscoelasticity for efficient soccer balls (Fig. 12).

### 3.5 Cycling helmets

Before a few years ago, plastics in bicycles were only used in a few applications. They were only used to make rubber brake blocks, handlebar grips, and tires. A small amount of plastic was used for lighting, saddles, cable covers, mudguards, and other items.^109^ The main components of a bicycle were all made of metal. In 1966, a bicycle built entirely of composite polymer materials was produced.^103,111^ The seat pillar and rear arm frame of this bicycle were two parts made of polyester that had been joined with glass fiber.^108^ Another bicycle, made of thermoplastic polyurethane (PU) and strengthened with glass and carbon fibers, was unveiled almost simultaneously.^108^ Composite materials are quickly replacing metal in the bulk of bicycle parts used today. Composite polymers enhance the comfort, weight, and strength of bicycles.^123,125^ Additionally, polymers are used in a wide range of sports and leisure pursuits.

**Fig. 13:**
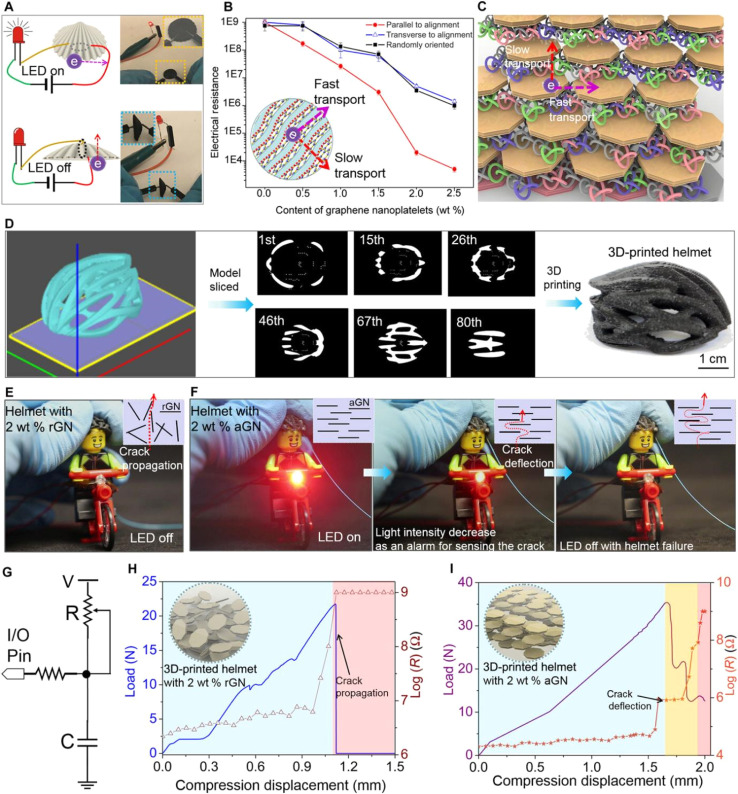
3D-printed smart helmet with anisotropic electrical property. (A) Anisotropic electrical property of the 3D-printed nacre. (B) Changes of electrical resistance with different GNs loadings and alignments. (C) Schematic diagram showing the layered polymer/GNs structure with anisotropic electrical resistance. (D) 3D-printing process of a self-sensing smart helmet. Demonstration of the wearable sensor on a Lego bicycle rider showing different self-sensing properties for the 3D-printed helmets with rGNs (E) and aGNs (F). (G) Circuit design for the tests. The compression force of the 3D-printed helmets with related compression displacements and resistance changes for rGNs (H) and aGNs (I), respectively.^178^ Copyright 2019, Science.

For instance, synthetic fiberboards, plastic dominoes, and plastic dart flights are all widespread in sports clubs. Food and soft drinks are frequently offered at sporting events on plastic forks, plates, and glasses.^125,143^ Polymers are used to create chess pieces, chips, counters, dice, and a variety of children's toys. Utilizing polymers reduces weight by a large amount, making things easier to transport and use.^97^ Cycling helmets' outer shells are frequently composed of impact-resistant polymers like polycarbonate.^90^ To lessen head injuries, this material distributes forces and offers impact protection.^108,109^ Expanded polystyrene (EPS) foam is frequently utilized within helmets to absorb and disperse impact energy. As a result of the foam's ability to compress upon impact, less force is applied to the skull. Yang *et al.*^178^ reported an electrically assisted 3D printing of polymers with the self-sensing capability of helmets, as illustrated in Fig. 13. To guarantee a snug fit and proper positioning on the head, helmets frequently have adjustable straps and retention systems composed of nylon or other tough polymers. Cycling helmets with ventilation systems sometimes have polymer-based vents that let air pass through, keeping the cyclist's head cool while they ride.^103,108,109,111^

## 4. Enhanced performance and safety

Improved performance and safety are crucial considerations in the design and production of sporting goods, particularly when it comes to polymers for sports applications.^47,53,59,64,68^ Polymers are adaptable substances that can be developed to have particular qualities that improve performance, sturdiness, and safety in a variety of sports-related items.^43^ In sports equipment, enhanced performance and safety polymers are used to produce lightweight design, impact resistance, energy absorption, flexibility, and elasticity.^31,44,45,53,59,64,179^ Certainly here is how this is done: here are some applications for improved performance and safety polymers in sporting goods.

### 4.1 Lightweight design

The definition of lightweight design is “the science and the art of making things, parts, products, and structures as light as possible, within constraints.” This mechanical interpretation only applies to items that must be rigid and robust enough to carry loads while remaining lightweight.^53,59,68^

• Polymers like sophisticated thermoplastics and carbon-fiber-reinforced composites are employed to make lightweight sporting equipment without sacrificing strength.

• Lightweight components in sports like cycling, where speed and maneuverability are important, require these materials.

• Lightweight polymers also help athletes exert less overall effort, which increases endurance and lessens tiredness.

The science and the art of making things, parts, products, and structures as light as possible, within constraints, is how lightweight design is defined. It is typically only used for objects that need to be stiff and strong enough to carry loads while still being lightweight, and this mechanical interpretation.^47,64^ Because it can be modified for prediction based on theory and for objective experimentation that produces repeatable outcomes, lightweight design is a science.^43,75^ For example, we can accurately estimate that, given an equivalent weight and 1.7 times the height and width of a steel beam, an aluminum solid square beam will be 70% stiffer than a steel one under bending.^48^ In addition, because it calls for personal and subjective judgment, intuition, and creativity, lightweight design is an art.

### 4.2 Impact resistance

Recent years have seen a rise in the use of high-performance materials in sportswear with increased usefulness. Impact protection is a feature of numerous sports-related products, including cycling, running shoes, rugby, football, and snowboarding apparel.^152,178^ There are few technical specifics regarding the various materials being used, and quantifiable indicators of their effectiveness are seldom disclosed.^152^ The use of such impact-resistant materials in sportswear has grown recently, although some customers have voiced concerns about how protection is built into the garments.^7,8,20^ Some clothing items include padding put inside fabric pouches that can shift during an impact and are unlikely to stay in place and shield the user from a fall, collision, or slip. Some clothing items have thick pads, which prevent the wearer's ability to breathe and limit their range of motion.^7,8,57^ However, the majority of clothing companies and sportswear companies assert that padding lowers the likelihood of sports-related injuries.^8^ Researchers contend, however, that such padding offers little defense against joint fracture and dislocation.^8,20,57^

• Protective equipment including helmets, pads, and guards are made of polymers with great impact resistance, such as polyurethane elastomers and designed thermoplastics.

• By absorbing and distributing impact pressures, these polymers reduce the risk of harm during accidents or falls.

• Sports equipment also uses improved impact-resistant polymers to avoid damage and retain performance even after extensive use.

### 4.3 Energy absorption

The utilization of polymer foams is growing in importance because of the potential weight reduction and value-added properties.^21,85,106^ Because of the unusual ability of foam structures to absorb energy, which is connected to particular deformation mechanisms of the cell structure (cell wall buckling and cell collapse), this field of study is very attractive.^55,167^ The sports business takes advantage of this property because the major goals of these items are to safeguard athletes' health and create secure playing environments.^16,21,55,85,103,106,167^

• Helmet liners and padding are made of energy-absorbing polymers like expanded polypropylene (EPP) and expanded polystyrene (EPS).

• By transforming kinetic energy into heat energy, these materials absorb impacts, lessening the force applied to the athlete's head or body.

### 4.4 Flexibility and elasticity

• Flexibility and elasticity are provided by polymer mixes and compounds in sports equipment such as gloves, boots, and clothing.^6,73,179^

• While maintaining strength and shape recovery, silicone-based polymers and thermoplastic elastomers (TPEs) offer flexibility.

• Elastic and flexible polymers improve comfort, dexterity, and ease of movement in footwear and gloves.

## 5. Future trends and innovations

Future developments and advances in sports goods polymers will most likely be influenced by a combination of variables like as sustainability, performance enhancement, and customization. Here are some potential paths the industry could take.

### 5.1 Eco-friendly materials

The use of environmentally friendly materials is having a big impact on the sports sector. With increased awareness about the impact of human activities on the environment, several sports businesses are turning to these materials for their goods.^47,53,59,68^ These materials, which range from recycled plastics and biodegradable materials to organic cotton and sustainable leather, are not only better for the environment, but they can also improve the performance and lifetime of sporting equipment.^31,44,64,179^ There are various advantages to utilizing eco-friendly materials in sporting equipment. For one thing, it aids in the reduction of waste and pollution, supporting sustainability and conserving the environment.^43,45,61^ Second, it can improve the performance and durability of sporting equipment. The use of environmentally friendly materials results in gear that is not only more comfortable but also more breathable, allowing athletes to stay cool and dry during exercises.^48^ These trends are changing the future of the sports sector and revolutionizing the way we engage and experience sports, from the usage of eco-friendly materials to the integration of wearable technology, personalization, augmented reality, and smart sports equipment.^73^

### 5.2 Smart sports goods

**Fig. 14:**
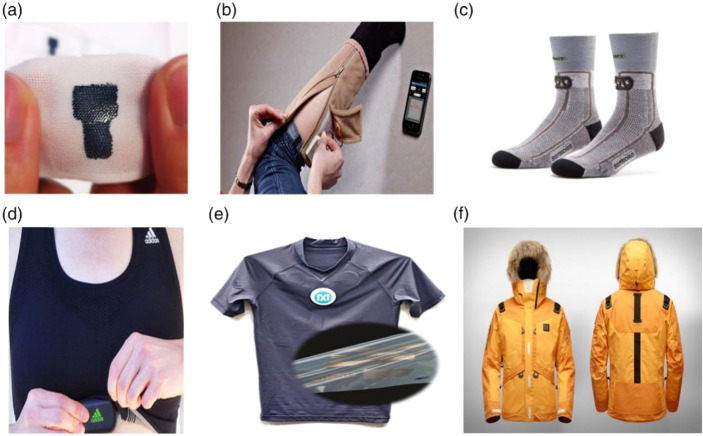
Role of polymers in smart sports goods especially health, and fitness applications: (a) textile wristband with a PEDOT:PSS electrode, (b) Edema stocking device, (c) sensoria smart socks, (d) Adidas textile electrode for sport top, (e) fitness shirt with biometric function, (f) life tech jacket.^179^ Copyright 2017, Sage.

Hundreds of sensors are utilized annually as a result of the growing use of sensors in the electronics, transportation, recreation, and other industries. Low-cost sensing is necessary as well, but it must perform better and have more features. Sports employ sensors in many different ways and for many different purposes.^73^ Simply by offering the most basic technology currently available to collect data from various sensors like accelerometers, GPS, gyroscopes, and other similar devices, numerous gadgets are accessible for a wide variety of sports.^48,68,179^ Among other things, sensors may be found in tennis balls, athletic equipment, footballs, skis, sports and football helmets, vests and outerwear, track shoes, and training shoes.^85,98,159,160,163^ The fourth industrial revolution, or “Industry 4.0”,^180^ is taking place right now. We have quickly developed into a very analytical culture where enormous datasets are constantly being created, gathered, and analyzed. Cities and nations seek to develop into intelligent societies that enhance the performance and welfare of their citizens. Everyday acts can easily be monitored and recorded. However, handling a massive volume of data is the most challenging. The sports business is comparable. Sports are a reflection of society. Patients, professionals—physical education teachers, coaches, analysts, doctors, therapists, *etc.*—as well as athletes of all levels, from amateur to world-class, are interested in learning about analytical metrics.^7,8,176^ Technology advancements can help athletes perform better, aid in performance analysis, and minimize and prevent sports injuries (Fig. 14).^2,5^

### 5.3 Customization and personalization

Potential for a new paradigm of product realization—the personalization of items catered to the distinct requirements and preferences of customers is presented by the accessibility of emerging responsive manufacturing methods, such as 3D printing, as well as by the pervasiveness of the internet and computing.^21,178^ To create innovative products and realize value, customers work together with producers and other customers. This co-design method is made possible by open product architecture. Involving users in product design, simulation/certification, production, supply, and assembly processes allows on-demand manufacturing systems and responsive cyber-physical systems to quickly satisfy client preferences.^32,45,47,48,61,64,66,179^ Consumers are involved in the design process at many different levels. Numerous designers are much more likely to be untrained individuals who bring a wide range of personal tastes and design methodologies with them. Research is required into the development and incorporation of new user interfaces that will assist both seasoned and beginning designers, as well as perhaps the professional design mentor as an interactive tool for the designer. Many consumers will gain a lot of knowledge while the design is being created.^74^

**Fig. 15:**
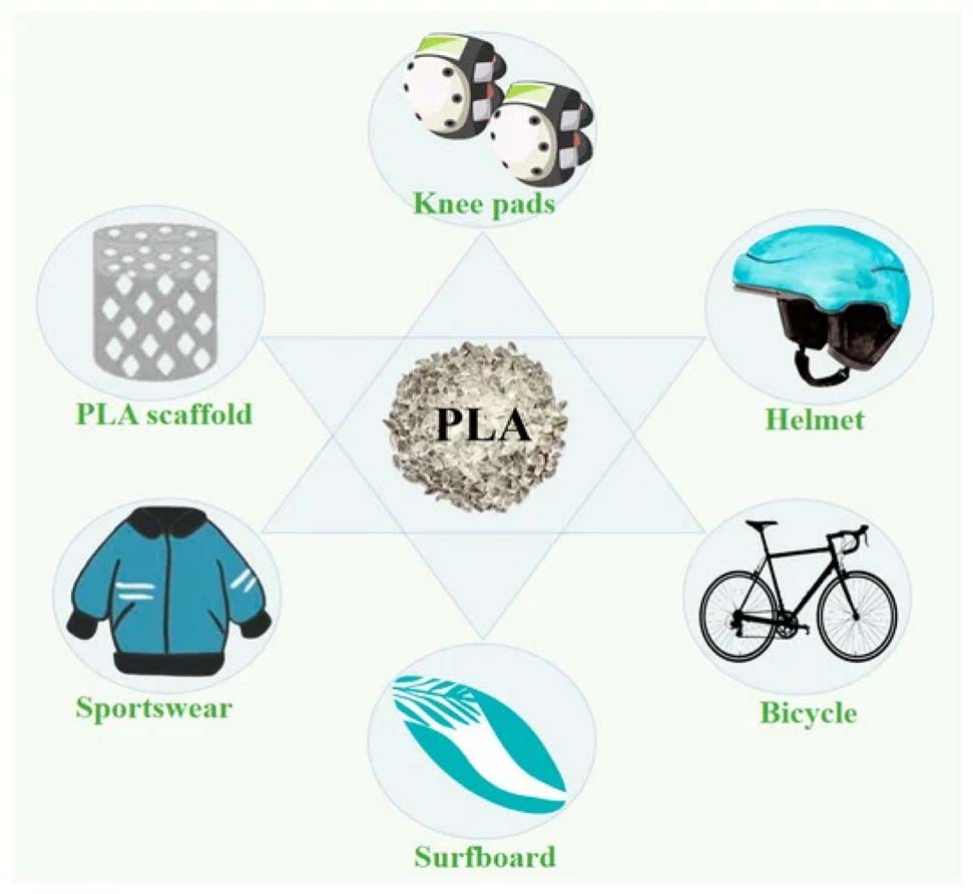
Polylactic acid applications in the sports industry.^61^ Copyright 2023, MDPI.

Customization and personalization are the processes of modifying products, services, experiences, or content to suit a person's preferences, requirements, and characteristics. These concepts have become more significant in a variety of fields, including technology, e-commerce, marketing, entertainment, and more.^31,43,44,66,68^ Customization and personalization aim to enhance user satisfaction, engagement, and overall experiences by offering content or goods that interact with people on a more personal level. Since the late 1990s, new technologies for producing polylactic acid (PLA) with a high molecular weight have increased its applications.^61^ Plastics made from PLA are increasingly being used in various fields, particularly the sports industry, as an alternative to synthetic polymers derived from petroleum (PETs, polystyrene (PS), polyethylene (PE), *etc.*), as illustrated in Fig. 15.^61^

Due to the industry's rising development and accessibility of more luxurious material products, customized and personalized products (CPP), which may best suit the needs of individual clients, are growing in popularity.^47^ The CPP paradigm is crucial for many firms to succeed in the fragmented, diverse, and competitive world. But to deliver CPP, manufacturers need a wider range of (and more) resources and capabilities, which would be prohibitively expensive in terms of building new facilities and hiring qualified personnel.^46^ Additionally, there might be significant fluctuations in those resources' and capabilities' utility rates over time, which can result in a lot of waste.^45^ They want things to happen, not the way the vendors are proposing. More and more, customers are looking for ways to offer value—for both the businesses/providers and for themselves.^45^

### 5.4 Wearable technology integration

Because research suggests that users are interested in these digital innovations for monitoring their surroundings, fitness, and health, wearable technologies such as fitness trackers like the Fitbit Charge and Garmin Vivo Sport and smartwatches like the Samsung Galaxy Watch and Apple Watch give teachers creative ways to teach.^47,59,66,74,129^ And are used to improve the processes of teaching and learning.

**Fig. 16:**
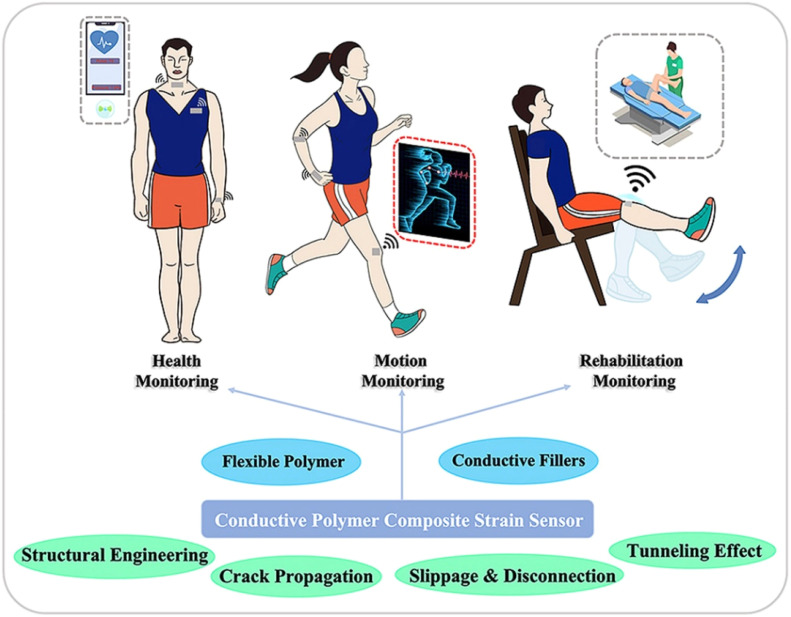
Wearable and stretchable conductive polymer composites for strain sensors.^73^ Copyright 2022, Elsevier.

Wearable technologies, sometimes known as wearable devices or just “wearables”, with their many looks, features, and uses, have come to represent modern technology.^73,84^ The Qing Dynasty in 17th-century China saw the invention of the first abacus ring.^181^ The first abacus ring was created in the 13th century in Italy, the pocket and wristwatch were created in the 16th century in Germany, and eyeglasses were invented in the 13th century in Italy.^43^ However, the concept of wearable technology has much deeper historical roots. These examples may not be “advanced technology” in the contemporary sense, but they are nevertheless essential to the design and capabilities of recent wearables. Lin *et al.*^73^ reported wearable and stretchable conductive polymer composites for strain sensors in sports wearable applications (Fig. 16).

### 5.5 Wearable technology, innovation and education

Despite wearable technology's increasing popularity recently, there hasn't been much research on its use in schools.^59,143^ You may make the case that due to a lack of research, the educational sector is unable to fully utilize wearable technologies for teaching and learning. One of four emerging technologies that have the potential to improve education and learning is wearable technology.^158^ The other three are adaptive learning, artificial intelligence, and learning analytics. In the educational arena, wearable technology offers a wide range of alternatives for teacher–student interaction, student engagement, contextual learning, evaluation, feedback, and more.^68,158^ It can also serve as a tool that is extremely portable, individual, discrete, common, useable, and intuitive in the framework of contextual learning theory and technology-modified lifelong learning theory.^68,158^ According to research, designs for wearable technology that promote fitness also have positive cognitive, behavioral, and affective effects, making them appropriate for use in a range of settings, such as the office and classroom.^158^ Incorporating wearable technology into theory-based course content is made possible by evidence that suggests positive associations exist between the use of wearable monitoring devices and frequency, intensity, and time (FIT) values.^158^

### 5.6 Indicators in sports monitoring

Utilizing methodical, scientific monitoring techniques to assess performance indicators in modern sports has arisen as a major issue that could improve player performance.^182^ The results can be used to modify movement and posture, monitor people's reactions in real-time, and quickly alter training schedules.^182^ The upshot is that the optimal schemes can boost competition, save unnecessary wear and tear, and avoid avoidable accidents. Flexible, wearable sensors have made it possible to monitor a wide range of signs.^182^

### 5.7 Kinematic indicators

**Fig. 17:**
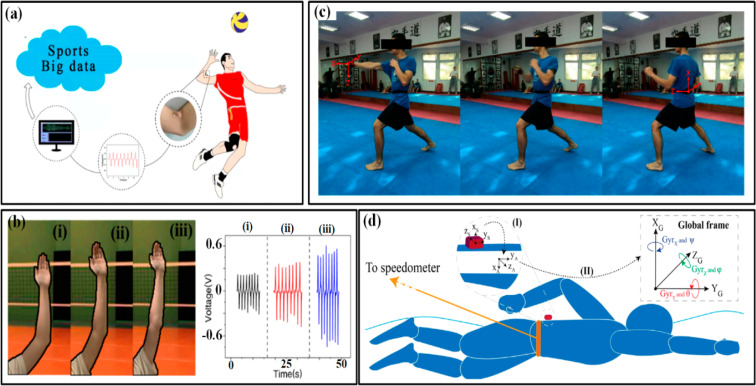
The kinematic markers in the trunk and limb are (a) the elbow bending angles in volleyball and (b) the observed outcomes of three elbow bending states (i–iii).^184^ Copyright 2021, MDPI. (c) Karate's use of IMUs. (d) Using IMUs while swimming.^185^ Copyright 2021, Frontiers in Bioengineering and Biotechnology.

A group of physical metrics known as kinematic indicators deal with the positions and movements of objects. First of all, posture more specifically, the deformation of bodily parts can describe the amplitude, direction, and frequency of motions.^1,58,183^ Effective monitoring can aid in identifying postural flaws, developing personal traits to improve training methods, and lowering injuries.^98^ For instance, the movement of the lower limbs can reveal a runner's stride frequency, step length, and knee/ankle joint angles, which helps analyze, assess, and then optimize the gait, step, and stride frequency strategies.^98^ Markovic *et al.*^184^ reported kinematic markers in the trunk and limb as illustrated in Fig. 17(a and b). Rad *et al.*^185^ also investigate the swimming techniques, as illustrated in Fig. 17(c and d).

### 5.8 Hand and foot

The hand is the most active component of our body, and numerous sports heavily rely on its movements. The throwing motions call for a well-balanced use of both hand and body motions, and the volleyball and handball hitting abilities are also directly correlated with hand shape.^60,89,119,123^ Tennis, ping pong, and badminton all benefit greatly from the monitoring of grip and exertion.^89^ The foot supports human motions and unavoidably endures intense pressure for an extended period.^3^ Variations in the contact area and plantar pressure provide a precise indication of the lower limb's stride and forcing mechanism.^159,161^ As a result, measuring and analyzing plantar characteristics is essential for athletes to evaluate their performance and avoid injuries. Finger bending and hand touch are the fundamental components of hand monitoring. Wearing flexible sensing strips on target fingers, whose movement creates bending/stretching stresses in the strips and subsequently results in a variation in their electrical properties makes it simple to monitor the bending of the finger or palm.^3^

**Fig. 18:**
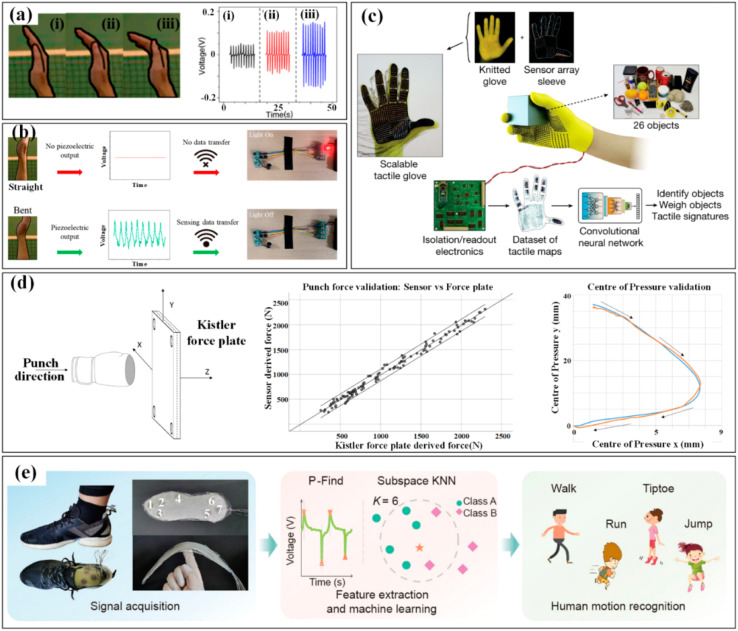
The three volleyball-related hand bending states (a) (i–iii) and the corresponding monitoring outcomes are among the kinematic indicators in the hand and foot. (b)The alarming system for hand gesture monitoring is among the indications in the hand.^186^ Copyright 2022, MDPI. (c) Tactile maps are produced by the knitted glove in the hand.^187^ Copyright 2019, Springer. (d) A piezoresistive pressure sensing system's measurement of the punch forces in boxing and a Kistler force plate's verification of those measurements.^188^ Copyright 2021, MDPI. (e) The distribution of plantar pressure to different motions as assessed by a printed insole.^189^ Copyright 2022, Springer.

The mechanical energy of athletes is converted into electricity using a flexible piezoelectric polyvinylidene difluoride (PVDF) film, which produces a voltage signal matching bending angles. Different angles of the palm can be identified from output voltages, as seen in Fig. 18a.^186^ The generated signal can be used to monitor posture and offer direction for volleyball players' daily training by fusing a wireless transmitting system and a big sports data platform (Fig. 18b).^186^ When it comes to hand contact, effective monitoring can be achieved by placing sensitive devices in the areas where gripping or touching takes place. To get the distribution of tactile forces, the units can either be grouped in an individual form at various interesting spots or merged into a sensor array. The latter strategy is more widely used, and numerous flexible, programmable smart gloves have been created to gather tactile data from the entire hand.

A common example is a low-cost, scalable tactile glove that has a variety of touch sensors covering the entire hand. Fig. 18c depicts the assembly of the 548-unit sensing array onto the surface of a knitted glove, which is utilized to produce distinct tactile maps when grabbing various things including Coke cans, balls, batteries, erasers, and others.^187^ It is possible to create tactile films with a frame rate of roughly 7.3 Hz using the obtained normal gripping motion forces, which range from 30 to 0.5 mm. The measurement and analysis of thorough punch metrics in boxing has also included wearable pressure monitoring. To quantify the punch forces, the developed piezoresistive pressure sensing system is incorporated into a 12-ounce boxing glove that has been approved by the International Amateur Boxing Association. A Kistler force plate is then utilized for verification (Fig. 18d).^188^ The sensing elements, which are often force and pressure sensors, can be individually placed in specific sections of the sole (such as the great toe, midfoot, metatarsal, and heel), or they can be put together into an array to cover the entire sole, depending on the required parameter. For instance, Yang *et al.* embedded seven sensing devices into a printed insole to track the body's distribution of plantar pressure and specific movements. Walking, running, tiptoeing, and jumping all result in dramatically different pressure distributions, as illustrated in Fig. 18e.^189^

## 6. Challenges and limitations

Let's explore the difficulties and restrictions related to cost and availability, toughness and longevity, and legal compliance for sports-related bio-inspired polymer products in more detail.

### 6.1 Cost and availability

#### 6.1.1 Costs of research and development

Creating bioinspired materials frequently requires a great deal of study, experimentation, and improvement. These procedures could be resource-intensive, which would raise the initial expenses of developing new materials.

#### 6.1.2 Obtaining raw materials

Specific or unusual raw materials that are not easily accessible may be needed for some bio-inspired products. These materials' sourcing may result in difficulties with the supply chain and higher expenses.

#### 6.1.3 Manufacturing methods

Compared to conventional materials, bio-inspired materials might require cutting-edge manufacturing methods or specialized machinery.

#### 6.1.4 Economies of scale

If the initial demand for bio-inspired materials is minimal, achieving economies of scale may be difficult. Costs can be gradually decreased with the aid of mass production and accessibility.

### 6.2 Durability and longevity

Sports equipment is put through a lot of use, which over time can cause wear and tear. It is vital to make sure that bio-inspired materials can preserve their functionality and structural integrity under these circumstances.

Long-term stability: over time, some bio-inspired materials may deteriorate or lose their qualities, which could reduce their durability. It might be difficult to create materials that maintain their performance qualities over time.

Outdoor sports equipment is subject to a variety of environmental factors, including dampness, UV rays, and changing weather. To resist these environmental variables without degrading, materials must be engineered.

### 6.3 Regulatory compliance

#### 6.3.1 Safety requirements

In order to protect athletes, sporting goods must adhere to safety requirements. To show that they adhere to these requirements, bio-inspired materials must undergo extensive testing.

#### 6.3.2 Sport-specific rules

To maintain fair competition, each sport has its own rules regarding gear and clothing. It can be difficult to ensure that bio-inspired materials adhere to these sport-specific standards.

Bio-inspired materials may introduce new substances or compounds, raising questions regarding their effects on both the environment and human health. It is crucial to adhere to the rules governing these matters. When these obstacles are overcome, the sports sector may be able to incorporate products made of bio-inspired polymers, providing athletes and fans with creative and sustainable alternatives.

#### 6.3.3 Biodegradable polymers

The emergence of biodegradable polymers as a viable biomaterial for food packaging applications has been facilitated by the growing concern regarding plastic pollution and environmental sustainability.^190–199^ These polymers are frequently produced from renewable resources, such as maize starch, potatoes, or sugarcane, and they offer a variety of advantages. When discarded, they decompose progressively into innocuous components, thereby reducing landfill waste and effectively protecting food products from environmental factors such as moisture and oxygen. Additionally, the increasing demand for environmentally favorable alternatives is met by food packaging that decomposes, which also extends the expiration life of food. However, there are still challenges, such as the achievement of the appropriate level of barrier characteristics, cost-effectiveness, scalability, and scaleability.

## 7. Conclusion

In conclusion, polymers play an indisputably major and revolutionary role in applications for sporting goods. By providing a wide choice of materials with remarkable qualities essential for boosting performance, safety, and durability in sports equipment and gear, polymers have revolutionized the sports sector. The adaptability of polymers allows manufacturers to customize the material properties to meet the demands of different sports, from lightweight and flexible polymers for comfortable sportswear to rigid and impact-resistant polymers for protective gear, enabling the development of products that cater to the specific requirements of each sport.

Athletes of all skill levels now have easier access to sporting equipment because of polymers, which provide an affordable alternative to more traditional materials like metal and wood. Athletes and athletic facilities benefit from longer use and lower maintenance costs thanks to polymers' durability and resistance to environmental variables.

Furthermore, the development of high-performance gear that improves athletic talents is a result of polymer composite innovation. For instance, carbon fiber-reinforced polymers have produced lighter and stronger gear, ultimately pushing the limits of human ability in a variety of sports.

Recognizing that there are drawbacks to the broad usage of polymers in sporting products, such as environmental issues with their manufacture and disposal is vital. In order to reduce the ecological impact of sporting goods, bio-based and biodegradable polymers are being developed as the sports sector works to become more sustainable.

In summary, polymers have changed the way sports goods are used by providing materials that are efficient, versatile, and specifically designed to meet the needs of players and sports aficionados. The role of polymers in sports is projected to further develop as sustainable practices and technology grow, ushering in a new era of innovation, performance, and environmental responsibility.

## Data availability

Data for this article are available and get permission from previous works.

## Conflicts of interest

There are no conflicts to declare.
